# Metagenomic analyses reveal previously unrecognized variation in the diets of sympatric Old World monkey species

**DOI:** 10.1371/journal.pone.0218245

**Published:** 2019-06-26

**Authors:** Martha M. Lyke, Anthony Di Fiore, Noah Fierer, Anne A. Madden, Joanna E. Lambert

**Affiliations:** 1 Department of Anthropology, University of Texas at San Antonio, San Antonio, TX, United States of America; 2 Department of Anthropology, University of Texas, Austin, TX, United States of America; 3 Department of Ecology and Evolutionary Biology and Cooperative Institute for Research in Environmental Sciences, University of Colorado, Boulder, CO, United States of America; 4 Department of Applied Ecology, North Carolina State University, Raleigh, NC, United States of America; 5 Department of Environmental Studies, University of Colorado, Boulder, CO, United States of America; East Carolina University, UNITED STATES

## Abstract

Insectivory, or the consumption of insects and other arthropods, is a significant yet cryptic component of omnivorous primate diets. Here, we used high-throughput DNA sequencing to identify arthropods from fecal DNA and assess variation in insectivory by closely-related sympatric primates. We identified arthropod prey taxa and tested the hypothesis that variation in insectivory facilitates niche differentiation and coexistence among closely-related species with high dietary overlap. We collected 233 fecal samples from redtail (*Cercopithecus ascanius*; n = 118) and blue monkeys (*C*. *mitis*; n = 115) and used a CO1 metabarcoding approach to identify arthropod DNA in each fecal sample. Arthropod DNA was detected in 99% of samples (N = 223 samples), and a total of 68 families (15 orders) were identified. Redtails consumed arthropods from 54 families, of which 12 (21.8%) were absent from blue monkey samples. Blue monkeys consumed arthropods from 56 families, of which 14 (24.6%) were absent from redtail samples. For both species, >97% of taxa present belonged to four orders (Araneae, Diptera, Hymenoptera, Lepidoptera). Redtail samples contained more Lepidoptera taxa (p<0.05), while blue monkey samples contained more Araneae (p<0.05). Blue monkeys consumed a greater diversity of arthropod taxa than redtail monkeys (p<0.05); however, the average number of arthropod families present per fecal sample was greater in the redtail monkey samples (p<0.05). These results indicate that while overlap exists in the arthropod portion of their diets, 20–25% of taxa consumed are unique to each group. Our findings suggest that variation in arthropod intake may help decrease dietary niche overlap and hence facilitate coexistence of closely-related primate species.

## Introduction

Understanding the mechanisms of species coexistence is an integral part of community ecology with implications for understanding species interactions at both evolutionary and ecological scales [1–3]. Classical niche theory states that when two or more similar species coexist, one or both must alter some aspect of their niche or one species will outcompete the other [4,5]. Resource competition has been implicated as an important component driving niche partitioning in coexisting species [4,5]. However, it is now widely recognized that, while competition can have a strong influence on species’ interactions, there may be other factors involved [1,3]. When two species evolve via allopatric speciation, divergent adaptations to their respective habitats can facilitate coexistence when the species come in contact [6]. For example, guenons (*Cercopithecus* spp.) are argued to have undergone allopatric speciation relatively recently (2.1–0.5mya) [7], and it is thought that divergent dietary adaptations have facilitated coexistence [8]. While there may also be direct resource competition, it is the differences in resource use–i.e., the areas of non-overlap–that are expected to enable species to coexist [3,6].

Species coexistence can be facilitated by differentiation in several niche parameters, including space (e.g., arboreal vs. terrestrial), time (e.g., diurnal vs. nocturnal), and diet (e.g., plant vs. animal). When closely-related species overlap in the spatial and temporal dimensions of resource use [3], such as with diurnal arboreal primates, differences in food resources are examined as a possible mechanism of niche differentiation and coexistence [4,6]. Indeed, aspects of plant food resource partitioning have been documented for a number of sympatric organisms, including many primate groups (e.g., *Lemur spp*. [9]; *Macaca* spp. [10]; *Cercopithecus* spp. [8,11]. However, because of methodological constraints, primate consumption of insects and other arthropods has been largely unexplored.

While few primate species are obligate insectivores (*sensu stricto*), most habitually consume some arthropods, and many are considered omnivorous [12–14]. Both human and nonhuman primates primarily consume arthropods from five main orders: Coleoptera, Hymenoptera, Isoptera, Lepidoptera, and Orthoptera [12–14]. Arthropods from these orders are attractive prey, as they are either social and found in localized high abundances (e.g., termite mound) or are slow moving during some life stages (e.g., caterpillars) making them vulnerable to exploitation [12]. Arthropods are generally high in proteins and lipids and contain essential micronutrients (e.g., vitamin B12) not present in plant foods [12]. But, while arthropods are nutrient dense, they are also relatively small and difficult to catch. Thus, based on metabolic energy requirements, only small-bodied (<500g) primates are predicted to obtain enough energy and nutrients to survive on a diet of solely arthropods [15]. Despite this prediction, arthropods are a substantial portion of the diets of several relatively large bodied (>4kg) primate species (e.g., *Erythrocebus patas pyrrhonotus*, [16]). Additionally, recent species-specific dietary analyses have reported habitual arthropod consumption in several primate species generally described as folivorous or frugivorous, including common chimpanzees (*Pan troglodytes*) [17], lowland gorillas (*Gorilla gorilla*) [17], l’Hoest’s monkeys (*C*. *lhoesti*) [18], bearded saki monkeys (*Chiropotes satanas chiropotes*) [19], woolly monkeys (*Lagothrix lagotricha poeppigii*) [20], and Hanuman langurs (*Presbytis entellus*) [21]. However, the proportion of the diet comprised of arthropods varies across the primate order, and the ecological and evolutionary impacts of omnivore arthropod consumption are not well known [12].

Guenons encompass a large and diverse monkey clade that often live sympatrically [7,22], making them ideal subjects for analyzing species coexistence. In Kibale National Park (KNP), blue and redtail monkeys are both diurnal, arboreal omnivores reported to have some dietary overlap [11,23]. Despite this overlap, there are important differences in the types and quantities of foods consumed [8,11,24–26]. For example, while both frequently consume fruit, blue monkeys tend to eat more leaves than redtails, and redtails consistently eat more arthropods [8,23]. Redtail monkeys have a smaller body size and are reported to consume arthropods with higher frequency at those sites where the two species live sympatrically [8,11,23,27]. Redtail monkeys are also reported to consume a greater diversity of arthropod species, including both slow and fast moving species [11,25], while blue monkeys have been observed to primarily consume slow moving species [11,25]. Previous observational research provides only broad parameters of insectivory in the study species at KNP, reporting that arthropods comprise a substantial portion of both blue (19.8–45.4%; [11,23,28]) and redtail monkey (21.8–55%; [8,11,23,24]) diets. However, despite their dietary importance, very little is known about the specific arthropod taxa consumed by these co-occurring monkey groups.

Gaining a complete understanding of insectivory has been challenging historically, as arthropod consumption is difficult to observe and arthropods themselves are often cryptic [29,30]. However, recent advances in genetic methods have made arthropod prey identification more practical, allowing for the evaluation of explicit hypotheses related to insectivory that were previously untestable [31–34]. Previous investigations of primate insectivory using DNA sequencing have provided proof of concept for this application of molecular methods using small sample sizes (n ≤ 20; [32,35,36], and in one larger study, Mallott et al. [37] sequenced the DNA of invertebrate prey of white-faced capuchins (*Cebus capucinus*) with the goal of investigating foraging strategies. However, here we present what is, to our knowledge, the first such analysis of primate insectivory with a sample size large enough to permit a robust analysis of variation between two closely related primate groups. We predicted that both blue and redtail monkeys would primarily consume taxa from the five most common orders (Coleoptera, Hymenoptera, Blattodea (Isoptera), Lepidoptera, Orthoptera), but the specific taxa consumed would vary. Based on previous observations, we also predicted that redtail monkeys would consume a greater diversity of arthropod taxa and their fecal samples would contain greater species richness than blue monkey samples. To test these predictions, we incorporated an integrative set of methods by combining field sample collection with recently developed molecular methods. Results of this research expand our knowledge of insectivory by omnivorous primates and have important implications for interpreting the role of insectivory in feeding niche differentiation and coexistence among closely related species.

## Materials and methods

### Study site

The field portion of this study was conducted in the Kanyawara site of the Kibale National Park (KNP), Uganda (0°13–0°19N and 30°19–30°32 E). KNP is in western Uganda near the foothills of the Ruwenzori Mountains. The park covers 766 km^2^ between the altitudes of approximately 1,200–1,500 m. KNP contains both primary and regenerating forest, ranging from mid-altitude moist evergreen to mid-altitude semideciduous [38,39]. Temperatures range from 12.7°C to 25.5°C, and annual rainfall is approximately 1,475mm with two reported rainy seasons, from March to April and October to November [39]. The Kanyawara study site contains a marked trail system allowing access to approximately 11 km^2^ of forest [38,39]. The study groups consisted of one habituated group each of redtail (*C*. *ascanius*) and blue (*C*. *mitis*) monkeys with overlapping home ranges inhabiting the Kanyawara site. GPS tracking during the study period showed that both groups utilized the same range throughout. At the time of our study, we could identify four males and twelve females in the redtail group and two males and eight females in the blue monkey groups. Subadults, juveniles, and infants were not sampled.

### Sample collection

Fecal samples were collected over a six-month period (July–December 2015) encompassing one dry–wet seasonal cycle. We collected a total of 233 fecal samples (redtails n = 118, blue monkeys n = 115). Intensive sampling (5–6 days per week) was conducted during the peak dry (July–August; n = 100) and peak wet (November–December; n = 88) months, and sampling was conducted one day per week during the transitional months (September–October; n = 49). Each study group was followed at least two days per week during intensive sampling and two days per month during transitional months. In general, we decided which group to follow on a given day by sampling need or first encounter. However, the two study species were often encountered together, as they spent at least some part of every sampling day foraging and traveling together.

We conducted full and partial day group follows and collected fecal samples opportunistically. Only fresh fecal samples were collected following an observed defecation event, and most samples were located and collected within 2–3 minutes of deposition. If we could not find a sample within 10 minutes, that sample was abandoned due to increased potential of environmental contamination. Only adult individuals were sampled, and the sex of the defecating individual was recorded. Fecal samples were placed directly into vials containing 95% ethanol with airtight seals and gently inverted to mix. Samples were labeled with the species name, sex, date, and time of defecation. After 24–36 hours in ethanol, samples were transferred to clean vials containing self-indicating silica desiccation beads to dry. Silica gel was checked and replaced daily until samples were completely dry. Vials were then sealed with parafilm and stored at room temperature for up to two months before being exported to the United States where they were then stored at -20°C until DNA extraction.

### Molecular analyses

DNA was extracted from all fecal samples using MoBio PowerFecal DNA Isolation kits following the manufacturer’s instructions. We used full plate PCR to amplify a ~157 bp region of the mitochondrial cytochrome c oxidase subunit 1 (CO1) gene using modified arthropod-specific primers [34,40]. Primers were modified to include unique adapters for sample identification following multiplex sequencing on the Illumina MiSeq platform, and the reverse primer for each sample was tagged with an individual error-correcting 12-basepair barcode. Error-correcting barcodes (e.g., Hamming codes) are used in massively parallel sequencing to reduce potential sequencing errors in large sample sets [41]. PCR amplification was performed in a total volume of 25 μL, including 3 μL gDNA, 12.5 μL Master Mix (Promega Biotech Co.), and 1 μM (1 μL) each of the forward and reverse primers. Cycling conditions followed Madden et al. (2016) (94°C for five min, followed by 45 cycles of 94°C 30 s, 45°C 45 s, 72°C 45 s and a final extension at 72°C for 10 min). PCR amplicons were visualized on 2% agarose gels, and 2–3 replicate PCR reactions were conducted per extracted DNA sample. Barcoded PCR products for each sample were pooled, cleaned, and normalized with SequalPrep Normalization Plates (Thermo Fisher Scientific). PCR products were then pooled across samples and sequenced on a single Illumina MiSeq run using 2x150bp paired-end chemistry at the University of Colorado BioFrontiers Institute Next-Gen Sequencing Core Facility. The standard amount of PhiX used during sequencing was increased from 15% to 30% to accommodate AT-rich amplicons as recommended by the manufacturer.

### Bioinformatics

Our multiplex sequencing run generated approximately 4.8 million sequences. Sequences were demultiplexed using a custom python script ‘prep_fastq_for_uparse.py’ (https://github.com/leffj/helper-code-for-uparse), and the reverse reads were used for downstream analyses due to higher quality scores. Sequences were quality filtered and operational taxonomic units (OTUs) were identified and clustered de novo at 99% similarity using the UPARSE pipeline (USEARCH v.7) [42]. Chimeric sequences were detected and removed, as were low quality reads (maxEE <1.50), short sequences (<32), and singletons (i.e. sequences that appeared only once across the dataset)[42]. Taxonomic assignments were made in QIIME [43] using the hierarchical naïve Bayesian classifier from the Ribosomal Database Project at 99% similarity and with a confidence of 0.5 [44] and a custom arthropod reference database curated from the Barcode of Life Database (v3) [45]. The molecular and bioinformatics pipeline used here was validated by Madden et al [40]. Where multiple arthropod OTUs were produced for a given family, unique OTUs were collapsed into their respective families for downstream analyses. All arthropod families identified were confirmed to occur on the African continent [46], though available data were insufficient to confirm presence more regionally (e.g., Uganda). After filtering, the average number of OTUs present per taxonomic family was 960 reads and the average number of OTUs per fecal sample was 783 reads. Following Madden et al.[40], any arthropod families represented by only a single OTU across all samples were conservatively removed after initial filtering, as were two OTUs present in one of the control samples. Sequences were then transformed into presence-only data, as sequence abundance can be skewed by variation in the size and digestibility of the individual arthropods consumed [40]. All sequences were submitted to the NCBI Sequence Read Archive (SRA) under accession number PRJNA521629.

### Data analysis

Species diversity indices were calculated using EstimateS software v9.1.0 [47], including rarefaction curves and Shannon diversity index. All other statistical analyses were performed using SPSS v23 (IBM) with significance set as p<0.05. We tested for normality in the number of OTUs per sample and the average diversity using the Shapiro-Wilk test and Q-Q plots. Where data were not normally distributed (species diversity data) we used non-parametric tests. Differences between the relative abundances of arthropod orders and families consumed by the two groups were calculated using Loglinear Poisson Generalized Linear Models. We tested for arthropod family richness per sample for each group using Independent t-tests and differences in the diversity of arthropod family DNA present using Mann-Whitney U tests.

### Compliance statement

Permission to conduct research for this project was granted by the Uganda Wildlife Authority and the Uganda National Council for Science and Technology. The research protocol was approved by the University of Texas at San Antonio’s Institutional Animal Care and Use Committee protocol # CE004-02/18. Our sampling methods did not require interactions with blue or redtail monkeys and did not manipulate the environment. No endangered or protected species or locations were involved in this study. All research conducted for this project was in compliance with the laws of Uganda.

## Results

Out of the 233 fecal samples in total, eight samples (one PCR plate column) failed to amplify, likely due to pipetting error, leaving 225 samples for downstream analyses (redtails n = 118, blues n = 107). Across these 225 fecal samples, we identified 15 arthropod orders, 68 families (Table 1), and 122 genera (S1 Table). Redtails consumed arthropods from 54 arthropod families in 11 orders (Table 1), of which 12 families (21.8%) and 4 orders (36.3%) were absent from blue monkey samples. Blue monkeys consumed arthropods from 56 families in 11 orders (Table 1), of which 14 families (24.6%) and 4 orders (36.3%) were absent from redtail samples.

**Table 1:** Arthropod families consumed by blue and redtail monkeys. X = present.

Order	Family	Common name	Blue	Redtail
Araneae	Araneidae	Orb-weavers	X	X
	Clubionidae	Sac spiders	X	X
	Eutichuridae	Prowling spiders	X	X
	Hahniidae	Dwarf sheet spiders	X	
	Linyphiidae	Sheet weavers	X	X
	Lycosidae	Wolf spiders	X	X
	Oxyopidae	Lynx spiders	X	X
	Philodromidae	Running crab spiders	X	X
	Pisauridae	Nursery web spiders	X	X
	Salticidae	Jumping spiders	X	X
	Theridiidae	Tangle-web spiders	X	X
	Thomisidae	Crab spiders	X	
Sarcoptiformes	Pyroglyphidae	Dust mites	X	
Scorpiones	Buthidae	Scorpions	X	
Trombidiformes	Eriophyidae	Galls	X	
	Eupodidae	Mites	X	
Entomobryomorpha	Entomobryidae	Springtails		X
Polydesmida	Paradoxosomatidae	Flat-backed millipedes		X
Coleoptera	Carabidae	Ground beetles	X	X
	Dermestidae	Skin beetles		X
	Phalacridae	Shining flower beetles		X
Diptera	Acroceridae	Small-headed flies	X	X
	Cecidomyiidae	Galls	X	X
	Chironomidae	Midges		X
	Culicidae	Mosquitoes	X	X
	Drosophilidae	Fruit flies	X	X
	Muscidae	True flies	X	
	Psychodidae	Sink flies		X
	Syrphidae	Hover flies	X	X
	Tabanidae	Horse flies		X
	Tachinidae	True flies	X	X
	Tephritidae	Fruit flies	X	X
Hemiptera	Acanthosomatidae	Shield bugs	X	
	Cicadidae	Cicadas	X	
	Miridae	Capsids	X	X
	Pentatomidae	Stink bugs	X	X
	Rhopalidae	Plant bugs	X	
Hymenoptera	Agaonidae	Fig wasps	X	X
	Braconidae	Parasitic wasps	X	X
	Eulophidae	Parasitic wasps	X	X
	Ichneumonidae	Parasitic wasps	X	X
	Perilampidae	Parasitic wasps	X	X
	Vespidae	Eusocial wasps	X	X
Lepidoptera	Crambidae	Grass moths	X	X
	Depressariidae	Moths	X	X
	Erebidae	Bright colored moths	X	X
	Eupterotidae	Moths	X	X
	Geometridae	Geometric moths	X	X
	Lasiocampidae	Snout moths	X	
	Limacododae	Slug moths	X	X
	Lycaenidae	Moths	X	X
	Noctuidae	Owlet moths	X	X
	Nolidae	Tuft moths		X
	Nymphalidae	Four-footed butterflies	X	X
	Oecophoridae	Concealer moths	X	X
	Papilionidae	Swallowtail butterflies	X	X
	Pieridae	Butterflies	X	
	Praydidae	Butterfllies	X	X
	Pyralidae	Snout moths	X	X
	Saturnidae	Large moths	X	X
	Sphingidae	Hawk moths	X	X
	Tortricidae	Leaf-roller moths	X	X
Mantodea	Mantidae	Praying mantis	X	
Neuroptera	Chrysopidae	Lacewings	X	X
Orhtoptera	Acrididae	Grasshoppers		X
	Gryllidae	Crickets		X
	Tettigoniidae	Katydids		X
Thysanoptera	Phlaeothripidae	Thrips		X

**Fig 1:**
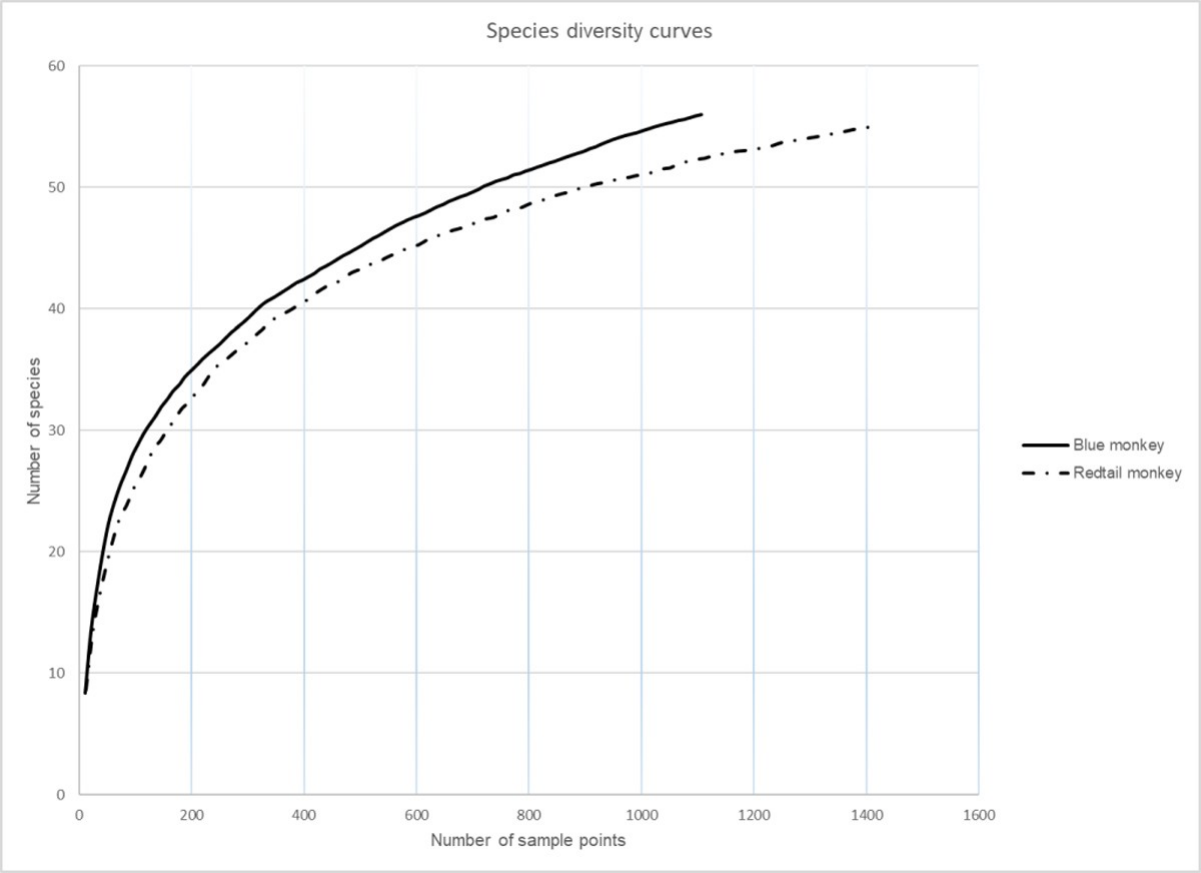
Species accumulation curves representing the arthropod families consumed by blue and redtail monkeys.

While it is difficult to quantify the proportion of diet comprised of arthropods, we could calculate the total number of arthropod families detected in each sample (family richness), allowing for a crude comparison of prey diversity between the species. The redtail monkey samples contained significantly more arthropod families per fecal sample (average = 12.1; range 0–25; t = -2.32, df = 221, p<0.05) than the blue monkey samples (average = 10.4; range 0–26). We also calculated the Shannon (H’) diversity index and plotted species accumulation curves for each primate species using EstimateS software ([47]; Fig 1). The blue monkey samples contained a significantly greater overall diversity of arthropod species (H’ = 3.17; U = 603.5, p<0.001) than the redtail samples (H’ = 2.91); however, both suggest high levels of diversity (H’ diversity mean > 2.5;[46]). Both species’ accumulation curves begin to asymptote (Fig 1), indicating that we have captured most of the likely prey diversity, but neither curve flattens completely, suggesting that further sampling might reveal even greater diversity.

**Fig 2:**
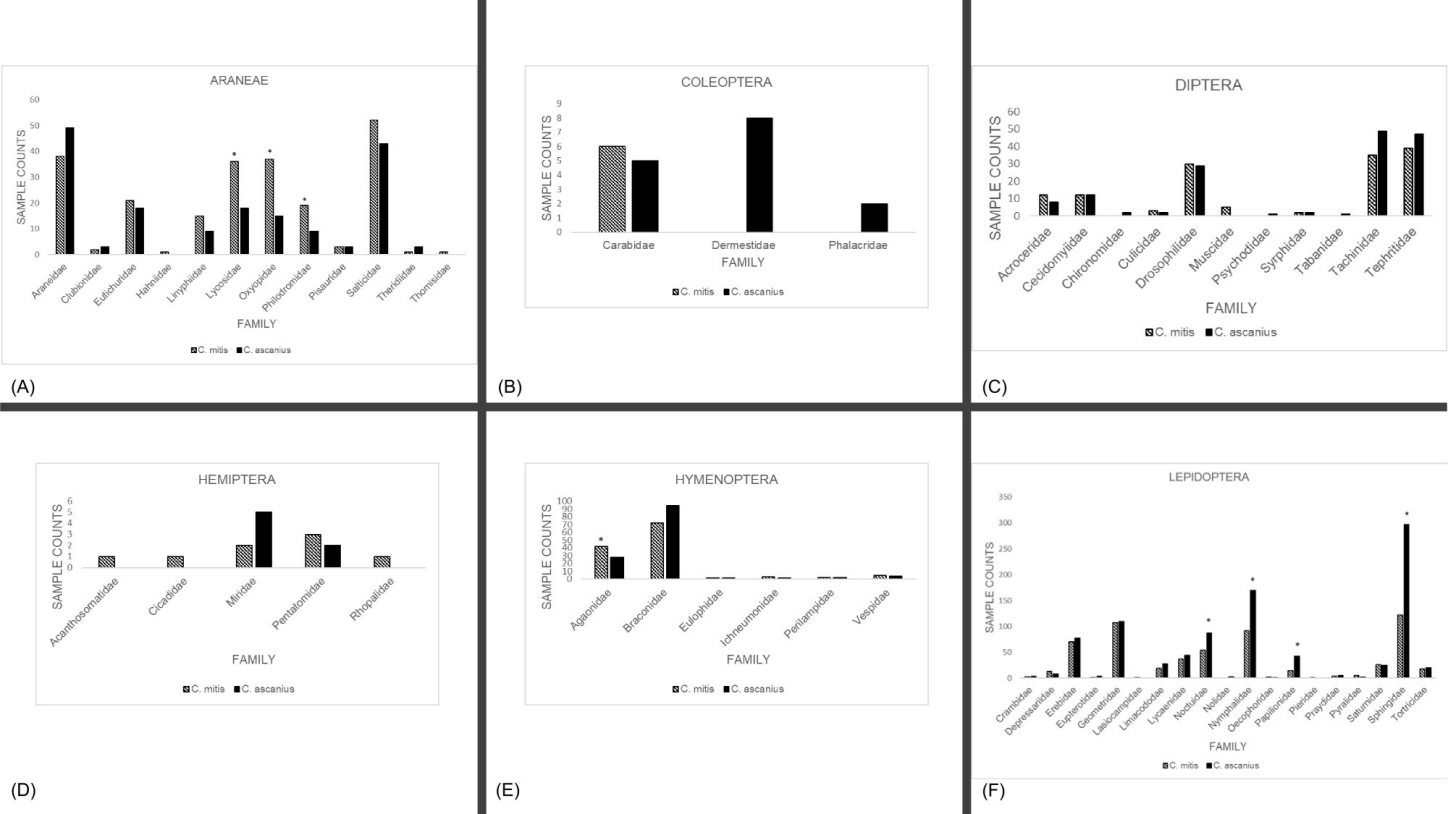
Total number of fecal samples where DNA was present by family for each of the primary orders. (A) Araneae. (B) Coleoptera. (C) Diptera. (D) Hemiptera. (E) Hymenoptera. (F) Lepidoptera. *p<0.05.

For both primates, more than 97% of the arthropod DNA detected was limited to four orders (Araneae, Diptera, Hymenoptera, Lepidoptera; Table 2), though the species differed in their relative consumption of these different orders. We calculated the percentage of the total taxa present for each of these four orders to estimate relative abundance. The largest variations were found in the relative abundances of DNA of Araneae (Table 2; *C*. *mitis* 20.43%, *C*. *ascanius* 12.02%) and Lepidoptera (*C*. *mitis* 53.25%, *C*. *ascanius* 65.56%). At the family level, there was variation in relative abundance within most orders (Fig 2), though overall variation was also only significant across Araneae (χ^2^ = 7.95, df = 1, p<0.01) and Lepidoptera (χ^2^ = 76.00, df = 1, p<0.001). We found significant variation between redtail and blue monkey samples in three Araneae families (Lycosidae, t = -3.12, p<0.05; Oxyopidae, t = -3.09, p<0.05; Philodromidae, t = -2.01, p<0.05; Fig 2A), one Hymenopteran family (Agaonidae, t = -2.42, p<0.05; Fig 2E), and four Lepidopteran families (Noctuidae, t = 2.11, p<0.05; Nymphalidae, t = 4.76, p<0.05; Papilionidae, t = 3.73, p<0.05; Sphingidae, t = 6.84, p<0.05; Fig 2F).

**Table 2:** Relative abundance (% of total present) of arthropod orders consumed by blue and redtail monkeys. The bolded orders represent the most abundant arthropod taxa detected. Asterisks indicate orders that were more commonly detected in one of the monkey species. *p<0.05.

		*C*. *mitis*	*C*. *ascanius*
Order	Common name	Relative abundance (%)	
**Araneae**	**Spiders**	20.43*	**12.02**
Sarcoptiformes	Mites	0.09	0
Scorpiones	Scorpions	0.09	0
Trombidiformes	Mites	0.18	0
Entomobryomorpha	Springtails	0	0.07
Polydesmida	Millipedes	0	0.14
Coleoptera	Beetles	0.54	1.06
**Diptera**	**Flies**	**12.48**	**10.82**
Hemiptera	True bugs	0.72	0.5
**Hymenoptera**	**Wasps, bees, ants**	**11.3**	**9.19**
**Lepidoptera**	**Moths, butterflies**	**53.25**	65.56*
Mantodea	Mantids	0.18	0
Neuroptera	Lacewings	0.72	0.14
Orthoptera	Katydids, grasshoppers, crickets	0	0.35
Thysanoptera	Thrips	0	0.14

### Caveats

One of the caveats of this study pertains to the small sample size of actual individuals and groups being sampled. Due to the costs and logistics associated with sample collection, export, preparation, and sequencing, we were constrained in the number of samples we were able to collect. Our goal was to collect a large number of samples from one group of each species to produce a robust analysis of the arthropod taxa consumed, which can be used as a baseline for future dietary analyses of the study species. Previous observations of the two study groups have provided information of the plant portion of their diets and shown that they frequently forage together. This makes them ideal subjects for our research focusing on niche differentiation among sympatric primates. While this limits us from making species-wide generalizations about arthropod consumption by blue and redtail monkeys, we provide an important first step towards a better understanding of guenon insectivory.

Additionally, some arthropod taxa may be underrepresented as an artifact of primer bias during the PCRs used to generate libraries for sequencing. In particular, the primer set we used is known to amplify Lepidopterans and Dipterans at a somewhat higher rate than other orders, and to under-amplify DNA from others orders, including Coleoptera and Isoptera [48]. Additionally, although the gene region we targeted (CO1) is frequently used for arthropod identification [48], some arthropod orders may not be well-represented in the reference database used here to identify OTUs [45]. Indeed, in the Barcode of Life database, there are only 2,025 records representing 295 species in Uganda, while there are 356,715 records representing 32,885 species in the United States. This general lack of reference DNA sequences has been reported for other tropical regions as well, and is one of the limitations to this type of study [32,36]. As a result, the putative taxonomic identities of the arthropods identified from the fecal samples should be considered with caution as the specific taxonomic identities cannot be confirmed with 100% certainty.

Another consideration pertains to the possibility of post-defecation environmental contamination. After defecation, fecal samples fall to the ground or onto leaves in the lower canopy, which exposes them to potential contaminants. Additionally, flies and other arthropods are attracted to and may lay eggs in feces, resulting in further DNA contamination. Here, fecal samples were collected as quickly as possible to reduce contamination risk, though some DNA contamination is inevitable.

## Discussion

In this study, we used high-throughput DNA sequencing to identify a large diversity of arthropod taxa consumed by sympatric guenons inhabiting KNP. As very few actual arthropod taxa have been identified previously (but see[49]), we present the first robust report of taxa consumed by these omnivorous primates. Because arthropods make up 20–50% of the diets of blue and redtail monkeys [23], these data represent an important component of their feeding ecology. We demonstrated that, while there was considerable overlap in arthropod prey, 20–25% of the arthropod taxa consumed were unique to each primate group. Specifically, we identified certain taxa (Table 1) that were more commonly found in the feces of one or the other monkey group, indicating some degree of arthropod prey specificity.

While we identified a wide diversity of arthropod taxa consumed, the most abundant orders only partially corresponded to our prediction that they would primarily consume Coleoptera, Hymenoptera, Blattodea (Isoptera), Lepidoptera, and Orthoptera. Lepidoptera was indeed the most abundant order consumed by both groups, but Araneae (spiders) was second most abundant, followed by Diptera (“true” flies) and Hymenoptera. While some groups may have been underrepresented in our results due to primer bias or lack of reference sequences, the high abundance of spiders in the diet of both groups was unexpected. Blue and redtail monkeys have not been observed to eat spiders [23,27,28,50]; however, a number of primate species are reported to consume spiders, including *Callicebus* spp. [32,51], *Cebus* spp. [37,52], *Galago* spp. [53], *Leontopithecus chrysomelas* [54], *Loris* spp. [55,56], *Macaca silenus* [57], *Saguinus* spp. [36,58], *Saimiri* spp. [32,59], and *Tarsius* spp. [56,60]. Some of the Araneae DNA identified is likely present in feces due to secondary ingestion of spider webs [61], but many of the Araneae families consumed do not weave webs (though all produce silk) [46]. Of the total samples where Araneae DNA was present, for blue monkeys 72% (n = 148) and for redtails 57% (n = 88) were families of spiders that do not build webs. This suggests that for these taxa, the monkeys are intentionally consuming spiders. Additionally, blue monkey samples contained more DNA (p<0.05) from the Lycosidae, Oxyopidae, and Philodromidae families, which are all fast-moving cursorial spiders, suggesting they may be more adept at catching fast-moving prey. This variation between the two monkey groups further indicates dietary and behavioral differentiation.

As redtail monkeys habitually consume more arthropods and are reported to consume a greater diversity of arthropod species [11,25], we predicted that we would detect a greater diversity of arthropod taxa in the redtail samples. However, we found a significantly greater diversity of arthropod DNA in the feces of blue monkeys than in the samples from redtails. We also predicted that redtail samples would contain a greater family richness (families per sample) of arthropod DNA, and this prediction was supported here. These results suggest that redtails may consume a greater number of arthropods, as there is a higher presence of arthropod DNA per sample, while blue monkeys consume a greater diversity of taxa. Overall, our data indicate that both the blue and redtail monkey groups sampled consume a diverse array of arthropod taxa and, as such, might be considered arthropod generalists.

Data presented here not only add to our understanding of the overall feeding ecology of these two species but are also useful for further exploring their nutritional ecology. While nutritional analyses are beyond the scope of this study, the identification of arthropod prey will be relevant to future investigations focused on the nutrient compositions of the diets of these two groups. In a recent analysis of the nutritional contribution to the diet of adult female redtail monkeys in KNP, Bryer et al. analyzed samples from three arthropod orders (Homoptera, Lepidoptera, Orthoptera) observed to be consumed. They reported that arthropods are an important source of protein and energy for the redtail monkeys; however, they also stated that difficulties in arthropod prey identification limited their ability to conduct a full nutritional analysis [49]. The results and methods presented here can provide a more robust index of arthropods consumed by the two groups, including a greater taxonomic distinction to the family level.

Another important aspect of primate insectivory relates to secondary arthropod consumption. It is expected that some arthropod taxa are consumed because of their presence in or on another food resource rather than because they are consumed intentionally. It can be argued, for example, that parasitic wasps are generally secondarily ingested, and we indeed detected large diversity of parasitoid DNA in samples consumed by both primate groups. Some parasitoids, such as *Agaonidae* spp. (fig wasps), are often consumed while fruits are consumed. Other parasitoids, such as *Braconidae* spp., have larval arthropod hosts, so they may be secondarily consumed with other arthropods. Additionally, spider DNA is present in spider silk, so some of the spider DNA detected is likely an artifact of secondary web consumption associated with eating leaves or other plant parts [61]. However, as stated above, many of the spider families detected in our samples do not weave webs. While secondary consumption might be problematic in assessing direct arthropod predation, such foods nonetheless contribute to the overall nutrition of the animals consuming them. Thus, their identification is valuable to understanding the overall dietary ecology of these primate groups.

Understanding the mechanisms of species coexistence is a key area of inquiry in community ecology. Where closely related species with substantial niche overlap live sympatrically, such as is the case with most guenons, it is important to recognize those areas of nonoverlap that permit coexistence. Sympatric primates do this in multiple ways, including resource switching [8], differential use of canopy [62], and differential use of food resources [9,10]. Here we report variation in the arthropod taxa consumed by sympatric guenons and identify previously unreported food resources. Further research will examine seasonal, inter- and intragroup, and intersexual variation in arthropod consumption by these two primate groups. Results of this research not only contribute to our understanding of the dietary ecology of blue and redtail monkeys, but also have implications for investigating primate dietary and nutritional ecology more broadly. The methodologies used here show promise for more detailed dietary analyses than have previously been possible and can yield insight into the ecological and evolutionary importance of arthropods throughout primate evolution.

## Supporting information

S1 Table Arthropod taxa identified from *Cercopithecus ascanius* and *C*. *mitis* feces via DNA sequencing. (PDF)
