# Supplementary material for: Metagenomic analyses reveal previously unrecognized variation in the diets of sympatric Old World monkey species
Source: PLoS One. 2019 Jun 26;14(6):e0218245. doi: 10.1371/journal.pone.0218245 (PMC6594596; doi:10.1371/journal.pone.0218245)
Supplement: S1 Table — (PDF) [file pone.0218245.s001.pdf]

Table S1: Arthropod taxa identified from *Cercopithecus ascanius* and *C. mitis* feces via DNA sequencing.

Phylum	Class	Order	Family	Genus
Arthropoda	Arachnida	Araneae	Araneidae_	
Arthropoda	Arachnida	Araneae	Clubionidae_	Clubiona
Arthropoda	Arachnida	Araneae	Eutichuridae_	Cheiracanthium
Arthropoda	Arachnida	Araneae	Hahniidae_	Cryphoeca
Arthropoda	Arachnida	Araneae	Linyphiidae_	
Arthropoda	Arachnida	Araneae	Linyphiidae_	Agyneta
Arthropoda	Arachnida	Araneae	Lycosidae_	
Arthropoda	Arachnida	Araneae	Oxyopidae_	
Arthropoda	Arachnida	Araneae	Oxyopidae_	Oxyopes
Arthropoda	Arachnida	Araneae	Oxyopidae_	Peucetia
Arthropoda	Arachnida	Araneae	Philodromidae_	
Arthropoda	Arachnida	Araneae	Philodromidae_	Philodromus
Arthropoda	Arachnida	Araneae	Pisauridae_	
Arthropoda	Arachnida	Araneae	Salticidae_	
Arthropoda	Arachnida	Araneae	Salticidae_	Carrhotus
Arthropoda	Arachnida	Araneae	Salticidae_	Evarcha
Arthropoda	Arachnida	Araneae	Theridiidae_	
Arthropoda	Arachnida	Araneae	Theridiidae_	Parasteatoda
Arthropoda	Arachnida	Araneae	Thomisidae_	
Arthropoda	Arachnida	Sarcoptiformes	Pyroglyphidae_	Dermatophagoides
Arthropoda	Arachnida	Scorpiones	Buthidae_	Centruroides
Arthropoda	Arachnida	Trombidiformes	Eriophyidae_	Aceria
Arthropoda	Arachnida	Trombidiformes	Eupodidae_	Eupodes
Arthropoda	Collembola	Entomobryomorpha	Entomobryidae	Lepidocyrtus
Arthropoda	Diplopoda	Polydesmida	Paradoxosomatidae_	Oxidus
Arthropoda	Insecta	Coleoptera	Carabidae_Harpalinae	
Arthropoda	Insecta	Coleoptera	Carabidae_Harpalinae	Lebia
Arthropoda	Insecta	Coleoptera	Dermestidae_Megatominae	Anthrenus
Arthropoda	Insecta	Coleoptera	Phalacridae_	Olibrus
Arthropoda	Insecta	Diptera	Acroceridae_	Ogcodes
Arthropoda	Insecta	Diptera	Cecidomyiidae_	
Arthropoda	Insecta	Diptera	Cecidomyiidae_	Feltiella

Arthropoda	Insecta	Diptera	Cecidomyiidae_	Resseliella
Arthropoda	Insecta	Diptera	Chironomidae_Orthocladiinae	Cricotopus
Arthropoda	Insecta	Diptera	Culicidae_Culicinae	Aedes
Arthropoda	Insecta	Diptera	Culicidae_Culicinae	Culex
Arthropoda	Insecta	Diptera	Drosophilidae_	
Arthropoda	Insecta	Diptera	Drosophilidae_	Zaprionus
Arthropoda	Insecta	Diptera	Drosophilidae_Drosophilinae	Drosophila
Arthropoda	Insecta	Diptera	Muscidae_	Polietes
Arthropoda	Insecta	Diptera	Muscidae_Coenosiinae	
Arthropoda	Insecta	Diptera	Muscidae_Coenosiinae	Coenosia
Arthropoda	Insecta	Diptera	Muscidae_Muscinae	
Arthropoda	Insecta	Diptera	Muscidae_Muscinae	Musca
Arthropoda	Insecta	Diptera	Psychodidae_Psychodinae	Psychoda
Arthropoda	Insecta	Diptera	Syrphidae_Syrphinae	
Arthropoda	Insecta	Diptera	Syrphidae_Syrphinae	Melanostoma
Arthropoda	Insecta	Diptera	Tabanidae_Tabaninae	Hybomitra
Arthropoda	Insecta	Diptera	Tachinidae_Exoristinae	
Arthropoda	Insecta	Diptera	Tachinidae_Exoristinae	Hyphantrophaga
Arthropoda	Insecta	Diptera	Tachinidae_Exoristinae	Lespesia
Arthropoda	Insecta	Diptera	Tachinidae_Tachininae	Lypha
Arthropoda	Insecta	Diptera	Tephritidae_Dacinae	
Arthropoda	Insecta	Diptera	Tephritidae_Dacinae	Bactrocera
Arthropoda	Insecta	Diptera	Tephritidae_Dacinae	Carpophthoromyia
Arthropoda	Insecta	Diptera	Tephritidae_Dacinae	Ceratitis
Arthropoda	Insecta	Diptera	Tephritidae_Dacinae	Trirhithrum
Arthropoda	Insecta	Hemiptera	Acanthosomatidae	
Arthropoda	Insecta	Hemiptera	Cicadidae	
Arthropoda	Insecta	Hemiptera	Miridae_Mirinae	
Arthropoda	Insecta	Hemiptera	Miridae_Orthotylinae	
Arthropoda	Insecta	Hemiptera	Pentatomidae_Pentatominae	
Arthropoda	Insecta	Hemiptera	Pentatomidae_Pentatominae	Nezara
Arthropoda	Insecta	Hemiptera	Rhopalidae_Serinethinae	Boisea
Arthropoda	Insecta	Hymenoptera	Agaonidae_	
Arthropoda	Insecta	Hymenoptera	Agaonidae_	Elisabethiella

Arthropoda	Insecta	Hymenoptera	Agaonidae_	Kradibia
Arthropoda	Insecta	Hymenoptera	Braconidae_Agathidinae	
Arthropoda	Insecta	Hymenoptera	Braconidae_Aphidiinae	Lysiphlebus
Arthropoda	Insecta	Hymenoptera	Braconidae_Meteorinae	Meteorus
Arthropoda	Insecta	Hymenoptera	Braconidae_Microgastrinae	
Arthropoda	Insecta	Hymenoptera	Braconidae_Microgastrinae	Apanteles
Arthropoda	Insecta	Hymenoptera	Braconidae_Microgastrinae	Cotesia
Arthropoda	Insecta	Hymenoptera	Braconidae_Microgastrinae	Diolcogaster
Arthropoda	Insecta	Hymenoptera	Braconidae_Microgastrinae	Nyereria
Arthropoda	Insecta	Hymenoptera	Braconidae_Rogadinae	Aleiodes
Arthropoda	Insecta	Hymenoptera	Eulophidae_Eulophinae	
Arthropoda	Insecta	Hymenoptera	Eulophidae_Eulophinae	Euplectrus
Arthropoda	Insecta	Hymenoptera	Ichneumonidae	
Arthropoda	Insecta	Hymenoptera	Ichneumonidae_Mesochorinae	
Arthropoda	Insecta	Hymenoptera	Ichneumonidae_Mesochorinae	Mesochorus
Arthropoda	Insecta	Hymenoptera	Perilampidae_Perilampinae	
Arthropoda	Insecta	Hymenoptera	Perilampidae_Perilampinae	Perilampus
Arthropoda	Insecta	Hymenoptera	Vespidae_Polistinae	
Arthropoda	Insecta	Hymenoptera	Vespidae_Polistinae	Polistes
Arthropoda	Insecta	Lepidoptera	Crambidae_Cybalomiinae	Trichophysetis
Arthropoda	Insecta	Lepidoptera	Crambidae_Spilomelinae	
Arthropoda	Insecta	Lepidoptera	Crambidae_Spilomelinae	Palpita
Arthropoda	Insecta	Lepidoptera	Crambidae_Spilomelinae	Stemorrhages
Arthropoda	Insecta	Lepidoptera	Depressariidae_Stenomatinae	Antaeotricha
Arthropoda	Insecta	Lepidoptera	Erebidae_Aganainae	Phaegorista
Arthropoda	Insecta	Lepidoptera	Erebidae_Arctiinae	
Arthropoda	Insecta	Lepidoptera	Erebidae_Arctiinae	Amerila
Arthropoda	Insecta	Lepidoptera	Erebidae_Calpinae	Eudocima
Arthropoda	Insecta	Lepidoptera	Erebidae_Erebinae	
Arthropoda	Insecta	Lepidoptera	Erebidae_Erebinae	Catocala
Arthropoda	Insecta	Lepidoptera	Erebidae_Hypeninae	Hypena
Arthropoda	Insecta	Lepidoptera	Erebidae_Lymantriinae	
Arthropoda	Insecta	Lepidoptera	Erebidae_Scoliopteryginae	Gonitis
Arthropoda	Insecta	Lepidoptera	Eupterotidae_	Stenoglene

Arthropoda	Insecta	Lepidoptera	Geometridae_Ennominae	
Arthropoda	Insecta	Lepidoptera	Geometridae_Ennominae	Cleora
Arthropoda	Insecta	Lepidoptera	Geometridae_Geometrinae	
Arthropoda	Insecta	Lepidoptera	Geometridae_Geometrinae	Hypodoxa
Arthropoda	Insecta	Lepidoptera	Geometridae_Geometrinae	Pingasa
Arthropoda	Insecta	Lepidoptera	Geometridae_Larentiinae	
Arthropoda	Insecta	Lepidoptera	Geometridae_Larentiinae	Chloroclystis
Arthropoda	Insecta	Lepidoptera	Geometridae_Larentiinae	Eupithecia
Arthropoda	Insecta	Lepidoptera	Geometridae_Oenochrominae	Dichromodes
Arthropoda	Insecta	Lepidoptera	Geometridae_Sterrhinae	Idaea
Arthropoda	Insecta	Lepidoptera	Lasiocampidae_Pinarinae	Pachypasa
Arthropoda	Insecta	Lepidoptera	Limacodidae	
Arthropoda	Insecta	Lepidoptera	Limacodidae	Strigivenifera
Arthropoda	Insecta	Lepidoptera	Lycaenidae_Polyommatinae	
Arthropoda	Insecta	Lepidoptera	Lycaenidae_Theclinae	
Arthropoda	Insecta	Lepidoptera	Lycaenidae_Theclinae	Aphnaeus
Arthropoda	Insecta	Lepidoptera	Noctuidae_Acronictinae	
Arthropoda	Insecta	Lepidoptera	Noctuidae_Acronictinae	Acronicta
Arthropoda	Insecta	Lepidoptera	Noctuidae_Noctuinae	
Arthropoda	Insecta	Lepidoptera	Noctuidae_Noctuinae	Leucania
Arthropoda	Insecta	Lepidoptera	Noctuidae_Noctuinae	Peridroma
Arthropoda	Insecta	Lepidoptera	Noctuidae_Noctuinae	Spodoptera
Arthropoda	Insecta	Lepidoptera	Noctuidae_Plusiinae	
Arthropoda	Insecta	Lepidoptera	Nolidae_Chloephorinae	Earias
Arthropoda	Insecta	Lepidoptera	Nymphalidae_	Pseudoneptis
Arthropoda	Insecta	Lepidoptera	Nymphalidae_Biblidinae	
Arthropoda	Insecta	Lepidoptera	Nymphalidae_Charaxinae	
Arthropoda	Insecta	Lepidoptera	Nymphalidae_Charaxinae	Charaxes
Arthropoda	Insecta	Lepidoptera	Nymphalidae_Charaxinae	Euxanthe
Arthropoda	Insecta	Lepidoptera	Nymphalidae_Heliconiinae	Acraea
Arthropoda	Insecta	Lepidoptera	Nymphalidae_Limenitidinae	
Arthropoda	Insecta	Lepidoptera	Nymphalidae_Limenitidinae	Cymothoe
Arthropoda	Insecta	Lepidoptera	Nymphalidae_Limenitidinae	Euryphura
Arthropoda	Insecta	Lepidoptera	Nymphalidae_Limenitidinae	Neptis

Arthropoda	Insecta	Lepidoptera	Nymphalidae_Nymphalinae	Junonia
Arthropoda	Insecta	Lepidoptera	Oecophoridae_Oecophorinae	
Arthropoda	Insecta	Lepidoptera	Oecophoridae_Oecophorinae	Micropeteina
Arthropoda	Insecta	Lepidoptera	Papilionidae_Papilioninae	Papilio
Arthropoda	Insecta	Lepidoptera	Pieridae_Pierinae	Colotis
Arthropoda	Insecta	Lepidoptera	Praydidae_Praydinae	Prays
Arthropoda	Insecta	Lepidoptera	Pyralidae_Phycitinae	
Arthropoda	Insecta	Lepidoptera	Pyralidae_Phycitinae	Hypargyria
Arthropoda	Insecta	Lepidoptera	Saturniidae_Saturniinae	
Arthropoda	Insecta	Lepidoptera	Saturniidae_Saturniinae	Cirina
Arthropoda	Insecta	Lepidoptera	Saturniidae_Saturniinae	Goodia
Arthropoda	Insecta	Lepidoptera	Saturniidae_Saturniinae	Imbrasia
Arthropoda	Insecta	Lepidoptera	Saturniidae_Saturniinae	Lobobunaea
Arthropoda	Insecta	Lepidoptera	Saturniidae_Saturniinae	Pseudobunaea
Arthropoda	Insecta	Lepidoptera	Sphingidae_Macroglossinae	
Arthropoda	Insecta	Lepidoptera	Sphingidae_Macroglossinae	Chaerocina
Arthropoda	Insecta	Lepidoptera	Sphingidae_Macroglossinae	Euchloron
Arthropoda	Insecta	Lepidoptera	Sphingidae_Macroglossinae	Nephele
Arthropoda	Insecta	Lepidoptera	Sphingidae_Macroglossinae	Temnora
Arthropoda	Insecta	Lepidoptera	Sphingidae_Macroglossinae	Xylophanes
Arthropoda	Insecta	Lepidoptera	Sphingidae_Smerinthinae	
Arthropoda	Insecta	Lepidoptera	Sphingidae_Smerinthinae	Andriasa
Arthropoda	Insecta	Lepidoptera	Sphingidae_Smerinthinae	Chloroclanis
Arthropoda	Insecta	Lepidoptera	Sphingidae_Smerinthinae	Falcatula
Arthropoda	Insecta	Lepidoptera	Sphingidae_Smerinthinae	Lycosphingia
Arthropoda	Insecta	Lepidoptera	Sphingidae_Smerinthinae	Neopolyptychus
Arthropoda	Insecta	Lepidoptera	Sphingidae_Smerinthinae	Polyptychoides
Arthropoda	Insecta	Lepidoptera	Sphingidae_Smerinthinae	Polyptychus
Arthropoda	Insecta	Lepidoptera	Sphingidae_Smerinthinae	Pseudoclanis
Arthropoda	Insecta	Lepidoptera	Sphingidae_Sphinginae	
Arthropoda	Insecta	Lepidoptera	Sphingidae_Sphinginae	Coelonia
Arthropoda	Insecta	Lepidoptera	Sphingidae_Sphinginae	Coenotes
Arthropoda	Insecta	Lepidoptera	Sphingidae_Sphinginae	Poliana
Arthropoda	Insecta	Lepidoptera	Sphingidae_Sphinginae	Xanthopan

Arthropoda	Insecta	Lepidoptera	Tortricidae_Olethreutinae	
Arthropoda	Insecta	Lepidoptera	Tortricidae_Olethreutinae	Dracontogena
Arthropoda	Insecta	Lepidoptera	Tortricidae_Olethreutinae	Thylacogaster
Arthropoda	Insecta	Lepidoptera	Tortricidae_Tortricinae	Choristoneura
Arthropoda	Insecta	Mantodea	Mantidae_	
Arthropoda	Insecta	Neuroptera	Chrysopidae_	
Arthropoda	Insecta	Orthoptera	Acrididae_	
Arthropoda	Insecta	Orthoptera	Gryllidae_Gryllinae	Gryllus
Arthropoda	Insecta	Orthoptera	Tettigoniidae_	Ruspolia
Arthropoda	Insecta	Thysanoptera	Phlaeothripidae	Haplothrips
